# Study of the effect of Sn grain boundaries on IMC morphology in solid state inter-diffusion soldering

**DOI:** 10.1038/s41598-019-51179-9

**Published:** 2019-10-16

**Authors:** Lin Hou, Nele Moelans, Jaber Derakhshandeh, Ingrid De Wolf, Eric Beyne

**Affiliations:** 1 Department Materials Engineering, KU Leuven, Leuven, Belgium; 2 IMEC, Kapeldreef 75, 3001 Leuven, Belgium

**Keywords:** Metals and alloys, Computational methods

## Abstract

This paper reports on 3D phase field simulations of IMC growth in Co/Sn and Cu/Sn solder systems. In agreement with experimental micrographs, we obtain uniform growth of the CoSn_3_ phase in Co/Sn solder joints and a non-uniform wavy morphology for the Cu_6_Sn_5_ phase in Cu/Sn solder joints. Furthermore, simulations were performed to obtain an insight in the impact of Sn grain size, grain boundary versus bulk diffusion, IMC/Sn interface mobility and Sn grain boundary mobility on IMC morphology and growth kinetics. It is found that grain boundary diffusion in the IMC or Sn phase have a limited impact on the IMC evolution. A wavy IMC morphology is obtained in the simulations when the grain boundary mobility in the Sn phase is relatively large compared to the interface mobility for the IMC/Sn interface, while a uniform IMC morphology is obtained when the Sn grain boundary and IMC/Sn interface mobilities are comparable. For the wavy IMC morphology, a clear effect of the Sn grain size is observed, while for uniform IMC growth, the effect of the Sn grain size is negligible.

## Introduction

Since microbumps are a key component for the 3D integration technology in microelectronics, research on the interface reactions between liquid or solid Sn-based solder and Cu UBM (under bump metallization) has attracted large interest^1–3^. Currently, the presence of Kirkendall voids inside the Cu_3_Sn IMC (intermetallic compound) phase, UBM consumption issues, and volume shrinkage of the solder joint, still cause serious electrical and mechanical reliability issues^4,5^. In order to miniaturize the solder-based interconnects to finer pitches, which is required for future technology nodes, suitable metallurgical systems with high reliability have to be found. Co, Ni, and Cu/Ni (bilayer structure) have been explored as replacements of the conventional Cu UBM^6–9^. Several advantages such as no void formation, the formation of only a single IMC phase, less UBM consumption and better electro-migration properties have been reported for Co/Sn and Ni/Sn systems^10–12^. A profound insight in the interfacial reactions and material properties for these alternative systems that could possibly replace the traditional Cu UBM is required to extend the interconnect technology to finer pitches with high reliability. But this know-how is still lacking.

Phase field simulations, modeling the microstructure evolution of multi-phase and multi-grain structures, can be used to get a better understanding and to predict the microstructure evolution resulting from the reactions at the solder – UBM interface. The phase field method has been applied to gain insight in the microstructure evolution during various metallurgical processes, such as solidification, precipitation, soldering and coarsening phenomena^13–16^. Extensive research has also been reported using the phase field method to simulate the microstructure changes and phase formation as a consequence of solid-liquid and solid-solid interfacial reactions between Sn-based solder and Cu UBM^17–19^. Guan and Moelans^17^ developed an approach using phase field simulations to estimate the solubility range of IMCs and verified the model versus experimental data for Sn solder in contact with Cu UBM. Xiong *et al*.^18^ deduced, based on phase-field simulations, that a thicker interfacial Cu_3_Sn layer, a thinner interfacial Cu_6_Sn_5_ layer and a faster consumption rate of the Cu pad can be obtained by increasing the pad size of the microbump. Park and Arroyave^19^ reported a multi-phase phase-field model to investigate the effect of model parameters, such as grain boundary diffusion and interfacial energy, on the morphology and growth rate of the IMCs during solid-state aging. The results of the simulations indicated that high grain boundary diffusion leads to an increase in the Cu_6_Sn_5_ IMC layer thickness and a decrease of the Cu_3_Sn IMC layer thickness. Phase field simulations can thus help to gain insight and estimate material properties related to the growth rate, solubility range and morphology evolution of the IMCs formed at the solder – UBM interface, and, in this way, support the ongoing experimental efforts^20^.

**Figure 1:**
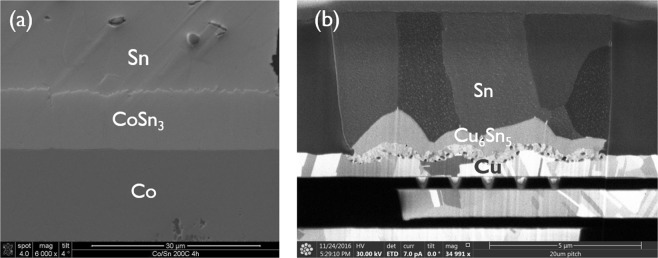
(**a**) Backscattered electron detector micrographs of the interfacial reactions of the Co substrate with Sn at 200 °C for 4 h. The formed IMC phase is CoSn_3_. (**b**) FIB images of a microbump with Cu UBM and Sn solder at 120 °C for 24 h.The formed IMC phases are Cu_6_Sn_5_ and Cu_3_Sn.

In all these studies, the effect of Sn grain size and Sn grain boundary properties during the solid-state aging process is still neglected and unclear, although recent experiments show a clear impact of the Sn grain structure on the morphology and growth kinetics of the Cu_6_Sn_5_ IMC layer^21^. Moreover, so far, no simulations have been performed for alternative systems besides the classical Cu-Sn joint, such as the Co-Sn based solder joints. Several studies reveal that the interface between the CoSn_3_ IMC phase and the Sn phase is relatively flat in Co-Sn couples, in contrast to the wavy and scalloped shaped Cu_6_Sn_5_ IMC/Sn interface after solid-state thermal treatment in Cu-Sn couples^22,23^. In this paper, similar observations are made for solid-state aging experiments of in-house fabricated samples, as shown in Fig. 1(a,b). This paper therefore reports a phase field simulation study for the Co/Sn system. The IMC evolution and morphology obtained for the Co/Sn system is compared with that obtained in Cu/Sn systems. In addition, the impact of Sn grain size, grain boundary versus bulk diffusion, IMC/Sn interface mobility and Sn grain boundary mobility on the IMC morphology and growth kinetics is simulated and discussed, providing insight in the different morphology obtained for Cu/Sn and Co/Sn systems.

## Phase Field Model

**Figure 2:**
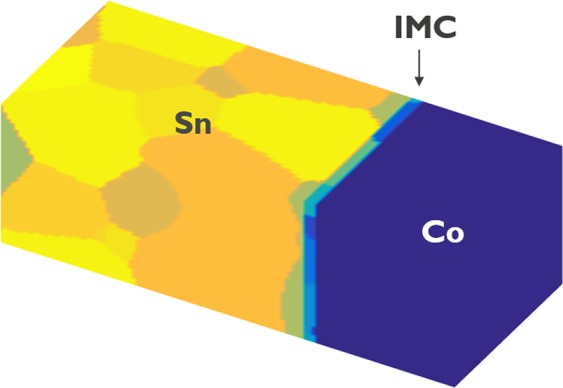
Schematic illustration of the computational domain considered in the Co/Sn simulations consisting of a solid solution UBM phase, the intermetallic compound (IMC), and a solid solution phase Bct-(Sn). Phases are represented with non-conserved order parameters *η*_*i*_ where the index *i* indicates the different grains.

This section describes the phase field model for multi-phase materials with different crystallographic orientations, i.e. grains, within each phase. Grains of different phases and different grain orientations are identified using non-conserved phase-field variables, *η*_*i*_ (*r*, *t*), which are continuous functions of space *r* and time *t*, with *i* = 1, …., *N*, $N={\sum }_{1}^{P}{p}_{\rho }$, and *p*_*ρ*_ is the number of grain orientations considered for phase *ρ* and the number of phases *P* considered in the simulated system. *η*_*i*_ (*r*, *t*) = *1* represents the presence of grain *i*, which belongs to a certain phase and has a certain grain orientation and *η*_*i*_ (*r*, *t*) = 0 represents the absence of this grain type at a certain point in the system. Diffuse interfaces are assumed in this model, which means that the phase-field variables of adjacent phases vary smoothly from 0 to 1 and vice versa at the grain boundaries and interfaces. Furthermore, a conserved field variable *x*_*Sn*_ (*r*, *t*) representing the local molar fraction of Sn is used to resolve the composition variations in space and time. A schematic illustration of the computational domain considered in the simulations is shown in Fig. 2.

The evolution of microstructure in an isothermal system is driven by minimization of the total Gibbs energy of the system when the pressure and temperature are constant values. In the present phase field simulation, the total Gibbs energy of a heterogenous isothermal system, *F*, consists of a bulk chemical energy contribution *F*_*bulk*_ (which defines the equilibrium composition of the phases) and an interfacial energy contribution *F*_*int*_:

1 $$\begin{array}{c}F={F}_{bulk}+{F}_{{int}}.\end{array}$$

Since only little information is available on the interface properties, all the interfaces are supposed to have similar properties in this simulation study. The interfacial part *F*_*int*_ is then formulated as^24^

2 $${F}_{int}=\int m{f}_{0}({\eta }_{1},{\eta }_{2},\ldots ,{\eta }_{N})+\frac{\kappa }{2}\mathop{\sum }\limits_{i=1}^{N}{({{\nabla }}_{i})}^{2}dV,$$

where the integral is taken over the volume *V* of the system and *f*_0_ is a fourth order polynomial of the order parameter fields with degenerate minima at (*1*, …, *i*, … *N*) = (*0*, …, ±*1*, …, *0*)

3 $${f}_{0}=\mathop{\sum }\limits_{i=1}^{N}(\frac{{{\rm{\eta }}}_{i}^{4}}{4}-\frac{{{\rm{\eta }}}_{i}^{2}}{2})+1.5\mathop{\sum }\limits_{i=1}^{N}\mathop{\sum }\limits_{j > 1}^{N}{{\rm{\eta }}}_{i}^{2}{{\rm{\eta }}}_{j}^{2}+\frac{1}{4}.$$

The model parameters *m* and *κ* are related to the interfacial free energy *σ* [J m^−2^] and diffuse interface width *l*_*int*_ [m]. The interfacial energy *σ* and diffuse interface widths *l*_*int*_ are defined as

4 $${\rm{\sigma }}=\frac{\sqrt{2}}{3}\sqrt{m\kappa }$$

and

5 $$\begin{array}{c}{l}_{int}=\sqrt{\frac{8}{m}}\end{array}$$

respectively^24^. The diffuse interface width, *l*_*int*_, is considered as a numerical parameter in this study and will be chosen in function of the grain size, i.e. as large as possible, but sufficiently small so that grains can still be resolved in the simulations.

The bulk energy part *F*_*bulk*_ is considered as an interpolation of the molar Gibbs energies of the different phases ${G}_{m}^{\rho }$ as a function of composition with respect to temperature *T*, divided by a constant coefficient *V*_*m*_ approximating the molar volume of the alloy

6 $${F}_{bulk}=\int {f}_{b}({\phi }_{{\rm{\rho }}},{x}_{Sn})dV=\int \mathop{\sum }\limits_{{\rm{\rho }}=1}^{P}{\varphi }_{{\rm{\rho }}}\,{f}^{{\rm{\rho }}}({x}^{{\rm{\rho }}})dV=\int \mathop{\sum }\limits_{{\rm{\rho }}=1}^{P}{\varphi }_{{\rm{\rho }}}\frac{{G}_{m\,}^{{\rm{\rho }}}({x}^{{\rm{\rho }}},T)}{{V}_{m}}dV$$

where ${\sum }_{{\rm{\rho }}=1}^{{\rm{P}}}\ast$ is taken over all phases, *ϕ*_*ρ*_ is the local phase fraction of phase *ρ*, which is obtained from the non-conserved phase field variables *i* as

7 $${\phi }_{\rho }=\frac{{\sum }_{i=1}^{{p}_{\rho }}\,{{\rm{\eta }}}_{i}^{2}}{{\sum }_{i=1}^{N}\,{{\rm{\eta }}}_{i}^{2}}$$

with ${\sum }_{i=1}^{N}\ast$ a sum over all phase-field variables belonging to all phases, and *x*^*ρ*^ are defined to be the phase compositions at each point^25,26^

8 $${x}_{Sn}=\mathop{\sum }\limits_{\rho =1}^{P}\,{\phi }_{\rho }{x}^{\rho }$$

and

9 $${\tilde{\mu }}^{\alpha }({x}^{\alpha })=\cdots ={\tilde{\mu }}^{\rho }({x}^{\rho })=\cdots ={\tilde{\mu }}^{P}({x}^{P})=\tilde{\mu }$$

with ${\tilde{\mu }}^{\rho }=\frac{\,\,\partial {G}_{m}^{\rho }}{\partial {x}^{\rho }},$ the diffusion potential of phase *ρ* at composition on *x*_*Sn*_ = *x*^*ρ*^. The equal diffusion potentials in local area and mass balance are required in the system for phase compositions.

Changes in molar volume with composition or among the different phases are neglected in this study, since appropriate treatment of volumetric effects would require including elastic and plastic contributions in the model, which would make the model much more complex, while they are expected to be less important than the chemical and diffusion effects for the considered phenomena.

The evolution of each non-conserved phase-field variable *η*_*i*_, *i* = *1* … *N*, follows a time-dependent Ginzburg–Landau equation

10 $$\frac{\partial {{\rm{\eta }}}_{i}}{\partial t}=-\,L({{\rm{\eta }}}_{1},\ldots ,{{\rm{\eta }}}_{N})\frac{\partial F({{\rm{\eta }}}_{1},\ldots ,{{\rm{\eta }}}_{N},{x}_{Sn})}{\partial {{\rm{\eta }}}_{i}}$$

where *L*(*η*_1_, …, *η*_*N*_) is of the form

11 $$L({{\rm{\eta }}}_{1},\ldots ,{{\rm{\eta }}}_{N})=\frac{{\sum }_{i=1}^{N}{\sum }_{j > i}^{N}\,{L}_{ij}{{\rm{\eta }}}_{i}^{2}{{\rm{\eta }}}_{j}^{2}}{{\sum }_{i=1}^{N}{\sum }_{j > i}^{N}{{\rm{\eta }}}_{i}^{2}{{\rm{\eta }}}_{j}^{2}}$$

and the *L*_*ij*_ [m^3^N^−1^ s^−1^] are related to the mobilities of grain boundaries and interfaces between different grains and phases. For interfaces between a grain of a different phase, *L*_*ij*_ = *L*_*αβ*_. Diffusion controlled growth will be obtained for^24^

12 $${L}_{ij}={L}_{int}={L}_{\alpha ,\beta }^{eq}=\frac{4m}{3\kappa {({x}_{{\rm{\alpha }}}^{eq}-{x}_{{\rm{\beta }}}^{eq})}^{2}}(\frac{{M}^{{\rm{\alpha }}}+{M}^{{\rm{\beta }}}}{2})$$

with *M*^*α*^ and *M*^*β*^ the diffusion mobilities in the phases α and β, and ${x}_{{\rm{\alpha }}}^{eq}$ and ${x}_{{\rm{\beta }}}^{eq}$ the equilibrium molar fractions of Sn in the phases α and β, when in equilibrium with each other^24^. For *L*_*α*,*β*_ < ${L}_{\alpha ,\beta }^{eq}$, growth will be in the mixed diffusion and interface reaction-controlled mode and it will become more and more interface reaction controlled for decreasing *L*_*α*,*β*_ value. For boundaries between grains of a same phase, *L*_*ij*_ is related to the grain boundary mobility *μ*_*gb*_ as^24^

13 $${L}_{ij}={L}_{gb}=\frac{4}{3}\frac{{\mu }_{gb}}{{l}_{int}}$$

Including bulk and grain boundary diffusion, the evolution of the conserved molar fraction field *x*_*Sn*_ follows^26^

14 $$\frac{\partial {x}_{Sn}}{\partial t}=\nabla \cdot [[\mathop{\sum }\limits_{\rho =1}^{P}{\phi }_{\rho }{M}^{\rho }+\mathop{\sum }\limits_{i=1}^{N}\mathop{\sum }\limits_{j > i}^{N}{M}_{gb}{\eta }_{i}^{2}{\eta }_{j}^{2}]\nabla \tilde{\mu }]$$

with *M*^*ρ*^ related to the bulk diffusion coefficient *D*^*ρ*^ of phase *ρ* as

15 $${M}^{\rho }=\frac{{D}^{\rho }}{{\partial }^{2}{f}^{\rho }/\partial {x}_{Sn}^{2}}$$

and *M*_*gb*_ related to the grain boundary diffusion coefficient *D*_*gb*_ as

16 $${M}_{gb}=3(\frac{{D}_{gb}}{{\partial }^{2}f/\partial {x}_{Sn}^{2}})(\frac{{\delta }_{gb}}{{l}_{int}})$$

with *l*_*int*_ the chosen width of the diffuse interface in the phase-field model and *δ*_*gb*_ the physical width of the grain boundary, i.e. of the order of 0.5 nm.

## Simulation Setup and Parameter Determination

The bulk energy density in phase-field models can be constructed as a function of composition and temperature using CALPHAD^27,28^ models and databases, giving the most detailed representation of the thermodynamic properties of the different phases^29^. As shown in the work of Guan and Moelans^17^, the use of a Gibbs energy with parabolic composition dependence fitted to the thermodynamic properties and phase diagrams has several advantages for the intended study, as the complexity and computational cost to evaluate the bulk contribution in the free energy is largely reduced. The physical behavior of the system will still be reproduced correctly, since the phase compositions are not expected to deviate much from their equilibrium compositions in the intended simulations. The thermodynamic coefficients extracted for the Co-Sn system from the CALPHAD thermodynamic databases^30^ and the equilibrium concentration of each phase according to the procedure described in Appendix A are given in Table 1. In a diffusion couple, the motion of one constituent causes a counter flow of the other constituent or of vacancies. An inter-diffusion coefficient describing the intermixing effect of the diffusion, D, is defined^31^. Wagner’s method^32^ was used to extract the inter-diffusion coefficients from concentration profiles of a Co-Sn solid state inter-diffusion couple. The detailed discussion of the determination of the interdiffusion coefficients D for the different phases in a Co-Sn couple is given in Appendix B and the extracted values are listed in Table 1.

**Table 1:** The thermodynamic coefficients in the parabolic Gibbs energy function as obtained from the thermodynamic database for each phase considered in the Co/Sn simulations at 473 K (see details in Appendix A).

Phases	*A* ^ρ^	*B* ^ρ^	*C* ^ρ^	*x*_0_^*ρ*^	Inter-diffusion D^ρ^
Co	1.92 × 10^12^	−1.54 × 10^9^	−1.54 × 10^9^	0.0258	1 × 10^25^
CoSn_3_	—	—	−3.18 × 10^10^	0.7500	4.7 × 10^14^
Sn	5.01 × 10^13^	−2.55 × 10^9^	−2.55 × 10^9^	0.9925	1.4 × 10^14^

Appropriate values for *A*^*CoSn3*^ and *B*^*CoSn3*^ will be determined such that the experimental growth rate of the IMC is reproduced in the simulations as described in section 4. The parameters listed in this table were used in all simulations for the Co/Sn system. The interdiffusion coefficients D^ρ^ where determined as explained in Appendix B.

A standard finite difference discretization with five-point stencil was applied to solve the time-dependent Ginzburg–Landau equations and diffusion equation using C++ programming language. MPI (Message Passing Interface) was used for parallelization. The boundary conditions used in the simulation domain borders are Neumann (or no-flux) boundary conditions. The values used in all simulations for the grid spacing ∆x, interfacial energy σ, diffuse interface thickness *l*_*int*_, model parameter *m*, model parameter *κ* and molar volume V_m_, are listed in Table 2. The diffuse interface width *l*_*int*_ is considered as a numerical parameter in this study. It is taken as large as possible, but so that all the grains are well resolved (hence smaller than the grain size). The grid spacing is selected such that *l*_*int*_ is 8*∆x to have a sufficient resolution of the phase-field profiles at the diffuse interfaces. It was verified that, for these parameter values, the selection of interfacial width has a very limited impact on the simulation results, as long as sufficient grid points are present over the interface, as was also discussed in the work of Park *et al*.^19^ and Moelans^20^. The model parameters *m* and *κ* in Eqs (4) and (5) were taken to be 3.75 × 10^6^ J m^−3^ and 3 × 10^−7^ J m^−1^ for all the interfaces, giving an interfacial energy σ equal to 0.5 J m^−2^ and diffuse interface width *l*_*int*_ equal to 8*10^−7^ m. The values of the parameters, such as the Gibbs energy parameters of the IMC phase *A*^*CoSn3*^, *B*^*CoSn3*^ and grain boundary diffusion parameters, are varied in the different simulations to study their effect on the growth rate and morphology of the IMC. The considered values will be specified in the relevant sections.

**Table 2:** Simulation parameters used for Co/Sn and Cu/Sn phase field simulations.

Items	Value
Grid space Δx	10^−7^ m
Interfacial energy σ	0.5 J m^−2^
Diffuse interfacial length *l*_*int*_	8*Δx
*m*	3.75 × 10^6^ J m^−3^
*κ*	3 × 10^−7^ J m^−1^
Molar volume V_m_	10^5^ m^3^ mol^−1^

The parameters listed in this table are taken the same in all simulations.

## Further Assessment of the CoSn_3_ Thermodynamic Parameters

For the further assessment of the parabolic Gibbs energy composition dependence of the CoSn_3_ phase, the phase-field 1-D simulation is applied to CoSn_3_ IMC evolution at the interface of Co/Sn diffusion couple at 473 K. These 1-D simulations were performed to optimize the input parameters for binary diffusion couple simulations, which will next be used in the 3-D simulations. In these simulations, there are three phases (two interfaces) present. Each of the phases is represented by one phase-field variable, which means that only one crystal orientation is considered for each phase in the 1-D simulation. The initial compositions of Co and Sn end phases were taken to be equilibrium concentrations of the phase for all simulations in the present work. For the CoSn_3_ IMC phase, the initial composition is taken equal to the stoichiometric composition. The simulations start from phase-field profiles with sharp interfaces. The sharp interface become diffuse within the first 200 time steps^17^.

The thermodynamic parameters *A*^*CoSn3*^ and *B*^*CoSn3*^ of the parabolic Gibbs energy of the CoSn_3_ IMC could not be extracted from the thermodynamic database. In this section, their values will be optimized to get the same growth rate of the IMC in the simulations as in the experiment. The time dependence of the IMC thickness follows a parabolic law:

17 $$d={d}_{0}+k{t}^{\frac{1}{2}}$$

where *d* [m] is the thicknesses of the IMC layer with respect to time t [s], *d*_*0*_ is the initial IMC thickness, and *k* is the IMC layer growth-rate coefficient [m/s^0.5^]. With the given experimentally determined value for the diffusion coefficient, the thermodynamic parameters *A*^*CoSn3*^ and *B*^*CoSn3*^ will be chosen so that the growth rate coefficient *k* obtained in the simulations is close to the value obtained from experimental results.

Simulations, were performed assuming different values for the *A*^*CoSn3*^ and *B*^*CoSn3*^ parameters, listed as Cases S0 to S5 in Table 3. The system size used for these simulations was [600 × 3 × 3] with grid spacing equal to 10^−7^ m, giving a system length of 60 μm. By evaluating the thickness of the CoSn_3_ IMC layer at different time steps, the growth coefficient *k* for steady-state IMC growth can be determined.

**Table 3:** The thermodynamic coefficients *A*^*CoSn3*^ and *B*^*CoSn3*^ used in the bulk Gibbs energy densities of CoSn_3_ IMC phase for different Cases and the corresponding IMC growth-rate *k* [m s^0.5^] of CoSn_3_ phase from simulation results.

	*A* ^*CoSn*3^	*B* ^*CoSn*3^	*k*[*ms*^0.5^]
Case S0	*A* ^*Sn*^	*B* ^*Sn*^	4.04 × 10^−8^
Case S1	*A* ^*Sn*^	0	4.04 × 10^−8^
Case S2	0.5*A*^*Sn*^	*B* ^*Sn*^	5.92 × 10^−8^
Case S3	0.35*A*^*Sn*^	*B* ^*Sn*^	6.74 × 10^−8^
Case S4	0.23*A*^*Sn*^	*B* ^*Sn*^	8.42 × 10^−8^
Case S5	0.1*A*^*Sn*^	*B* ^*Sn*^	13.7 × 10^−8^
Experimental	—	—	8.27 × 10^−8^

The coefficients for Case S4 give the best fit with the experimental growth rate and will be used further for the simulations for polycrystalline systems.

**Figure 3:**
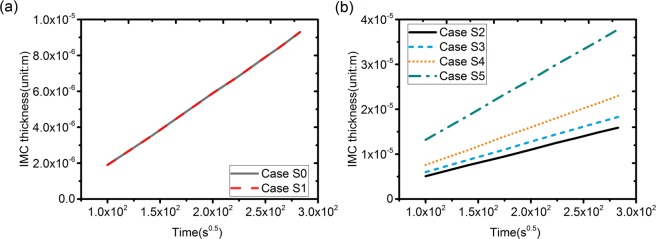
Layer thickness as a function of the square root of time of the IMC phases as obtained from the simulations for Case S1 to Case S5: (**a**) different *B*^*CoSn3*^ for Case S0 to S1; (**b**) different *A*^*CoSn3*^ for Case S2 to S5.

The layer thickness of the IMC phase as a function of the square root of time for Case S0 and S1 are shown in Fig. 3(a), which indicates that thermodynamic coefficients *B*^*ρ*^ seems to have a limited effect on the growth rates of the IMC phase. Therefore, for simplicity, *B*^*CoSn3*^ is taken equal to *B*^*Sn*^ for the Cases S2 to S5 to optimize the *A*^*CoSn3*^ parameters. The layer thickness of the IMC phase as a function of the square root of time for Case S2 to Case S5 with different values for *A*^*CoSn3*^ are shown in Fig. 3(b). It is clear that an increase in *A*^*CoSn3*^ in the parabolic free energy of the IMC phase results in a lower growth rate coefficient *k* implying a smaller solubility range of the IMC phase. The growth-rate coefficients of the CoSn_3_ IMC phase obtained from Case S0 to Case S5 with different thermo-dynamic parameters are listed in Table 3. Comparison with the experimental value of Takamatsu *et al*.^33^ (listed in Table 3), shows that the growth rate of the IMC has the best agreement with the experimental value for Case S4. The calculated growth-rate coefficient in the steady-state regime simulation is 8.42 × 10^−8^ m s^−0.5^. Moreover, for Case S4, it is calculated from Eq. (12) that diffusion-controlled growth is obtained in the simulations for the kinetic coefficient *L*_*int*_ equal to 2.78 × 10^−9^ m s for the IMC/Sn interface.

## 3-D Simulation of Solid-State Interfacial Reactions in Multi-Grain Structures

The thermodynamic coefficients of the parabolic free energy density functions listed in Table 1 and those listed for Case S4 in Table 3 are next used in the more complex 3D Co/Sn multi-grain simulations. The same inter-diffusion coefficients of the three phases (as listed in Table 1) are taken for these multi-grain simulations. For the Sn and the CoSn_3_ IMC phases, a multi-grain structure is assumed with isotropic grain boundary properties as described in Fig. 2. A simulation domain of size 120 × 50 × 50 grid points with grid spacing equal to 10^−7^ m is taken for all simulations. To generate the initial grain structure of the individual phases, a standard grain growth simulation assuming isotropic grain boundary properties is performed, considering different simulation times to obtain grain structures with a different initial size. It is important to mention that in the present work the nucleation of new grains at the interface is neglected. The impact from nucleation might affect the IMC growth and evaluation behavior but is out of the scope of the present work. The grain size is varied for the Sn phase and the impact of this Sn grain size will be discussed in this section as well. The effect of the kinetic coefficient *L*_*int*_ related to the mobility between different phases, the grain boundary diffusion parameter *M*_*gb*_ (which is related to the grain boundary diffusion coefficient *D*_*gb*_) and the coefficient *L*_*gb*_, which is related to the grain coarsening rate within the phases, will be considered in the multi-grain simulations. The ratio between *L*_*int*_ and *L*_*gb*_ is an important parameter and is defined as *α*

18 $$\alpha =\frac{{L}_{int}}{{L}_{gb}}$$

### Impact of grain boundary diffusion M_gb_

The importance of grain boundary diffusion is believed by several studies to be the major factor affecting the IMC morphology^34,35^. However, there is no simulation or theoretical work yet studying the impact from the Sn grain structure in combination with that of grain boundary diffusion. To investigate the effect of diffusion along the grain boundary paths in the Sn and IMC phases, three values of the grain boundary diffusion mobility *M*_*gb*_ will be considered in the simulations (listed in Table 4). The grain boundary diffusion mobility is always assumed to be equal in the Sn and IMC phase. Case M3 with *M*_*gb*_ equal to 0, is considered as the reference case, where there is only bulk diffusion. For the simulations presented in this section, *L*_*int*_ is taken equal to 2.78 × 10^−9^ m s, resulting in diffusion controlled growth of the IMC phase, as derived from Eq. (12) for the optimized Case S4. No experimental data related to the grain boundary mobility for Sn (Co) are available to determine the value of *L*_*gb*_. However, it was experienced that when *L*_*gb*_ < *L*_*int*_ is taken in the simulations, the grain boundaries in the IMC and Sn phase cannot follow the movement of the interfaces between the different phases, resulting in artificial effects in the simulations. Therefore, *L*_*gb*_ = *L*_*int*_ is taken in all simulations to minimize the effect of grain coarsening and still obtaining physically meaningful simulation results.

**Table 4:** The grain boundary diffusion mobility *M*_*gb*_, *L*_*int*_ (related to the mobility of the interfaces between different phases) and *L*_*gb*_ (related to grain boundary mobility and hence the grain coarsening rate) used in Case M0 to Case M3.

	*M*_*gb*_ [m^2^ mol J^−1^ s^−1^]	*L*_*int*_ [m^3^ N^−1^ s^−1^]	*L*_*gb*_ [m^3^ N^−1^ s^−1^]
Case M0	2.36 × 10^−23^	2.78 × 10^−9^	2.78 × 10^−9^
Case M1	2.36 × 10^−24^	2.78 × 10^−9^	2.78 × 10^−9^
Case M2	2.36 × 10^−25^	2.78 × 10^−9^	2.78 × 10^−9^
Case M3	0	2.78 × 10^−9^	2.78 × 10^−9^

**Figure 4:**
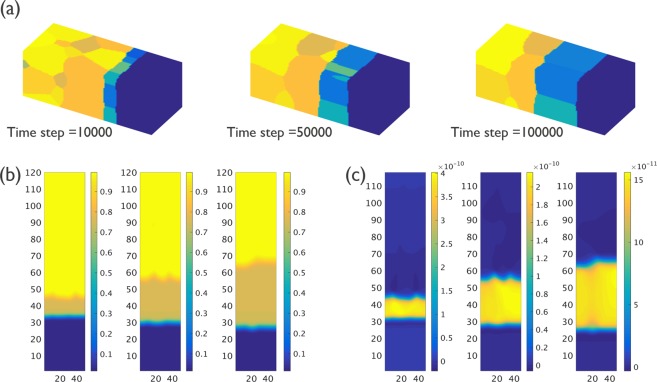
(**a**) 3-D view of IMC evolution for Case M0 from time step 10000 to 100000 (for α = *L*_*int*_*/L*_*gb*_ = 1); different grains are represented by different colors; (**b**) The cross-section composition of IMC evolution as obtained from the phase-field simulation; (**c**) The cross-section image of the vertical flux as obtained from the phase-field simulation.

The simulation results of the IMC evolution of Case M0, with the highest grain boundary diffusion *M*_*gb*_, from time step 10000, 50000, 100000 are shown in Fig. 4(a). A rather uniform IMC morphology is observed. Cross-section views of the concentration and vertical diffusion flux as obtained in the phase field simulations are shown in Fig. 4(b,c), respectively. The IMC thickness is slightly higher near the Sn grain boundaries, but overall, the IMC thickness is rather uniform. The vertical diffusion flux is also almost uniform inside the IMC phase.

**Figure 5:**
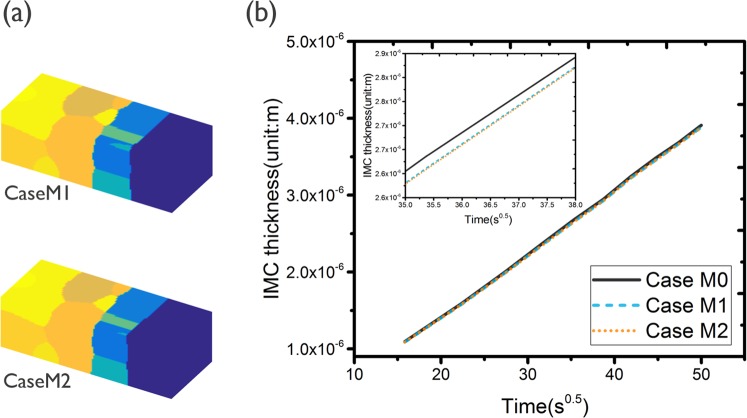
(**a**) 3-D view of IMC evolution for Case M1 and M2 (α = *L*_*int*_*/L*_*gb*_ = 1) for time step 50000; different grains are represented by different colors; (**b**) Layer thickness as a function of the square root of time of the IMC phases as obtained from the simulations for Case M0 to Case M2. The simulation result indicate that higher grain boundary diffusion results in thicker IMC layer. However, the impact from grain boundary diffusion *M*_*gb*_ is very limited.

Three-dimensional views of the grain structures for cases M1 and M2, with a different grain boundary diffusion mobility *M*_*gb*_, at time step 50000 are given in Fig. 5(a). The growth kinetics of the IMC layer for Case M0 to M2 are compared in Fig. 5(b). The thickness of the IMC layer is taken as the average thickness of the IMC phase over the simulation domain, which is calculated by summing the local volume fractions over all grid points, multiplied with the volume of a grid point, divided by the cross-section area of the whole simulated system. Compared with reference simulation Case M3 (with *M*_*gb*_ = 0, the result is shown in Appendix C Fig. A.3), the results shown in Fig. 5(a,b) indicate that grain boundary diffusion has very limited impact on the IMC morphology and growth rate coefficient in the considered simulations. This flat morphology is in agreement with the experimental findings for Co/Sn, as shown in Fig. 1(a). However, for the Cu/Sn system, a more wavy interface is found experimentally, which is usually devoted to effects from grain boundary diffusion *M*_*gb*_, whereas in the current simulations, a flat interface is found even in the presence of high grain boundary diffusion *M*_*gb*_. To understand which parameters, besides the grain boundary diffusion mobility *M*_*gb*_, affect the IMC morphology, further simulations studying the effect of the kinetics of the interfaces and grain boundaries, controlled by *L*_*int*_ and *L*_*gb*_ respectively, were performed and are discussed in the following sections.

### Impact of the relative magnitudes of interfacial and grain boundary mobility

To investigate how interfacial mobility and grain boundary mobility affect the IMC evolution, simulations were carried out with various input values for *L*_*int*_ for the IMC/Sn interface. It needs to be mentioned that in this section the aim is no longer to reproduce the experimental data for the Co/Sn system, but to study the effect of *L*_*int*_, which is related to the interface mobility, on the IMC morphology (Case M4 to M11, see Table 5). If *L*_*int*_ is lowered, the IMC growth rate will be changed and will no longer match the experimental kinetic growth rate. The other parameters, such as *M*_*gb*_ and *L*_*gb*_, have the same values as in Case M0 to M3. Again, different values for grain boundary diffusion mobility *M*_*gb*_ are considered to figure out the impact from grain boundary diffusion on the IMC morphology and growth kinetics with decreased *L*_*int*_. The ratio between *L*_*in*t_ and *L*_*gb*_ is given in Table 5 as α. For the Cases M0-M3 discussed in the previous section, α was equal to 1, while lower values are used in Case M4-M11.

**Table 5:** The grain boundary diffusion mobility *M*_*gb*_, kinetic coefficients *L*_*int*_ (related to interfacial mobility), *L*_*gb*_ (related to grain coarsening rate) and ratio α used in Case M4 to Case M11.

	*M*_*gb*_	*L*_*int*_	*L*_*gb*_	α = L_int_/L_gb_
Case M4	2.36 × 10^−23^	2.78 × 10^−10^	2.78 × 10^−9^	0.1
Case M5	2.36 × 10^−24^	2.78 × 10^−10^	2.78 × 10^−9^	
Case M6	2.36 × 10^−25^	2.78 × 10^−10^	2.78 × 10^−9^	
Case M7	0	2.78 × 10^−10^	2.78 × 10^−9^	
Case M8	2.36 × 10^−23^	2.78 × 10^−11^	2.78 × 10^−9^	0.01
Case M9	2.36 × 10^−24^	2.78 × 10^−11^	2.78 × 10^−9^	
Case M10	2.36 × 10^−25^	2.78 × 10^−11^	2.78 × 10^−9^	
Case M11	0	2.78 × 10^−11^	2.78 × 10^−9^	

**Figure 6:**
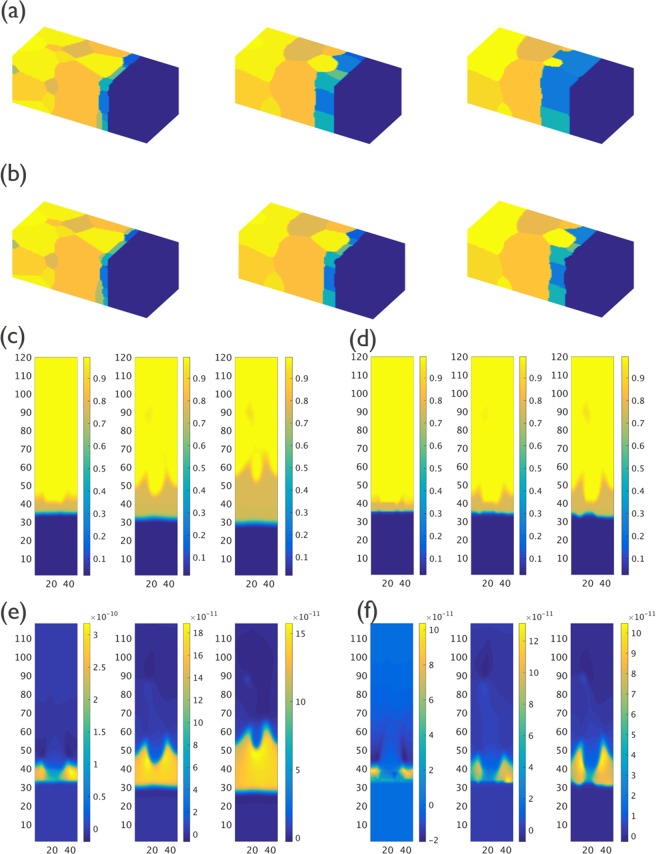
(**a**) 3-D view of IMC evolution for Case M4 (α = *L*_*int*_*/L*_*gb*_ = 0.1) for time steps 10000, 50000, 100000; (**b**) 3-D view of IMC evolution for Case M8 (α = *L*_*int*_*/L*_*gb*_ = 0.01) for time steps 10000, 50000, 100000; (**c**) The cross-section composition of IMC evolution for Case M4; (**d**) The cross-section composition of IMC evolution for Case M8; (**e**) The cross-section image of flux in vertical direction for Case M4; (**f**) The cross-section image of flux in vertical direction for Case M8.

The IMC evolution results of Cases M4 and M8, at time steps 10000, 50000 and 100000, are shown in Fig. 6(a,b), respectively. The cross-section views of concentration and flux along the vertical direction as obtained from the phase field simulations at different time steps are shown in Fig. 6(c,d) for Case M4, and in Fig. 6(e,f) for Case M8. Comparison with the simulation results for Case M1 clearly indicates the impact of the ratio α. In Cases M4 and M8, a considerably thicker IMC layer is found at locations around the Sn grain boundaries. It also can be seen that the thickness of the IMC layer in Case M8 is significantly smaller than for Case M4, due to the lower value for *L*_*int*_. The cross-section view of the flux along the vertical direction for Cases M4 and M8, as shown in Fig. 6(d,f), also indicate that the diffusion is not uniform. Compared with locations without Sn grain boundaries, the diffusion through the IMC phase at locations near a grain boundary in the Sn phase is faster when the ratio α < 1.

The IMC layer thickness as a function of the square root of time was found to be very similar for Case M4 and M7. The same results were found from the simulation results for Case M8 to Case M11. No obvious impact from the grain boundary diffusion mobility *M*_*gb*_ on IMC morphology and evolution behavior is thus found. Therefore, the three-dimensional views of the IMC evolution are not shown here but the simulation results of Case M11 (without grain boundary diffusion) are shown in Appendix C.

The simulations thus indicate that the IMC phase grows preferentially along the Sn grain boundaries when the Sn grain boundaries are more mobile than the IMC/Sn interface. Due to the higher grain boundary mobility, the structure allows more easily local IMC growth resulting in an irregular interface. The magnitude of the grain boundary diffusion seems to have little effect on this.

### Impact of Sn grain size and number

**Figure 7:**
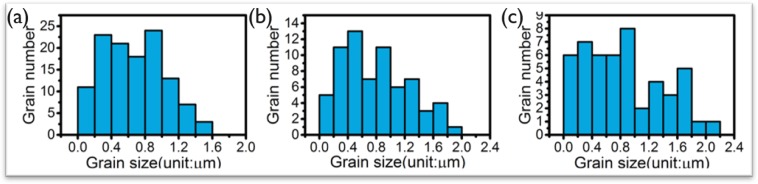
Grain size distribution of the three Sn grain structures used in the simulations listed in Table 7. (**a**) Average grain diameter is 0.8 µm; (**b**) Average grain diameter is 1.0 µm; (**c**) Average grain diameter is 1.1 µm.

The simulation results discussed in the previous section indicate that the Sn grain boundaries, their location and number, can have a large effect on the IMC morphology and growth rate. To investigate the effect of Sn grain size on the IMC morphology and kinetic growth rate during solid state reaction, simulations were carried out using Sn grain structures with different average grain size. The considered grain size distributions are shown in Fig. 7. The simulations presented in the previous sections 5.1–5.2 were performed with the grain structure with grain size distribution shown in Fig. 7(c) for the Sn phase, which is the one with the largest grain size. The different cases discussed in this section are listed in Table 6. Since the IMC evolution shows a significant difference with the ratio α = *L*_*int*_*/L*_*gb*_, the Sn grain size effect is investigated for two sets of simulations with a different input value for the ratio *α*. Cases M0 and M8, the ones discussed in sections 5.1 and 5.2, are considered as reference cases to study the effect of Sn grain structure.

**Table 6:** The grain boundary diffusion mobility *M*_*gb*_ (related to grain boundary diffusion), the kinetic coefficients *L*_*int*_ (related to interface mobility), the kinetic coefficient *L*_*gb*_ (related to grain boundary mobility) and the ratio α used in the simulation groups 1 and 2 to study the effect of Sn grain size on IMC evolution.

Group	Case	*M*_*GB*_	*L*_*int*_	*L*_*GB*_	α	Sn grain size (see Fig. 7)
Group 1	Case M0	2.36 × 10^−23^	2.78 × 10^−9^	2.78 × 10^−9^	1	(c)
	Case M12	2.36 × 10^−23^	2.78 × 10^−9^	2.78 × 10^−9^		(b)
	Case M13	2.36 × 10^−23^	2.78 × 10^−9^	2.78 × 10^−9^		(a)
Group 2	Case M8	2.36 × 10^−23^	2.78 × 10^−11^	2.78 × 10^−9^	0.01	(c)
	Case M14	2.36 × 10^−23^	2.78 × 10^−11^	2.78 × 10^−9^		(b)
	Case M15	2.36 × 10^−23^	2.78 × 10^−11^	2.78 × 10^−9^		(a)

**Figure 8:**
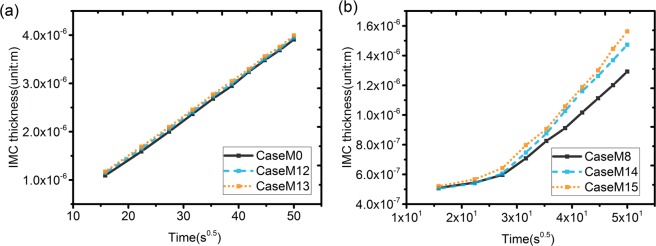
(**a**) Layer thickness as a function of the square root of time of the IMC phases as obtained for Case M0, M12 and M13 (α = *L*_*int*_*/L*_*gb*_ = 1); (**b**) Layer thickness as a function of the square root of time of the IMC phases as obtained for Case M8, M14 and M15 (α = *L*_*int*_*/L*_*gb*_ = 0.01). The result indicates a strong impact from Sn grain size.

**Figure 9:**
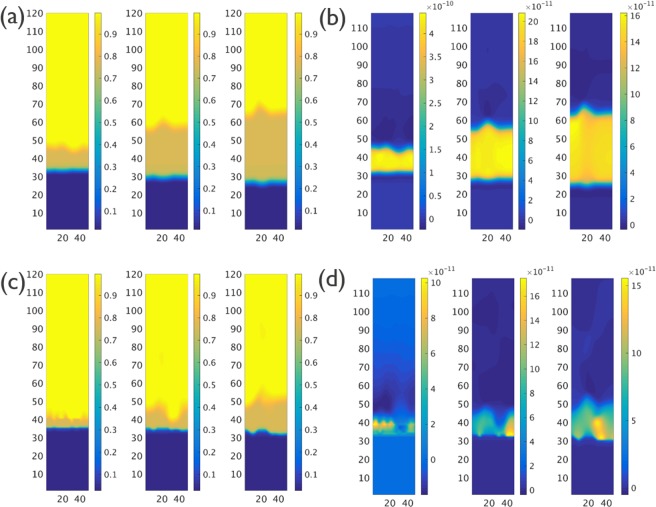
(**a**) The cross-section composition of IMC evolution as obtained from CaseM13; (**b**) The cross-section image of flux in vertical direction as obtained from CaseM13; (**c**) The cross-section composition of IMC evolution as obtained from CaseM15; (**d**) The cross-section image of flux in vertical direction as obtained from CaseM15.

The layer thickness of the IMC phase as a function of the square root of time as obtained for the Group 1 simulations (α = 1) are shown in Fig. 8(a). The impact of Sn grain size on the IMC growth kinetics is very limited for this group of simulations. The IMC evolution with time for Case M13, which has the smallest Sn grain size, is shown in Fig. 9(a). Similar to the Case M0, the IMC growth and vertical diffusion flux are uniform even though more grain boundary paths are introduced in the simulation. The layer thickness of the IMC phase as a function of the square root of time as obtained from the Group 2 (α = 0.01) simulations are shown in Fig. 8(b). Unlike the simulation results for Group 1, the growth of the IMC phase is influenced significantly by the Sn grain size. A larger growth rate is observed for smaller Sn grain size. The simulation results for Case M15, which are shown in Fig. 9(c) reveal a clear effect of the number and position of grain boundaries in the Sn phase on the IMC morphology. The diffusion flux along the vertical direction shown in Fig. 9(d) indicates a higher flux at the location near Sn grain boundaries. The impact of grain boundary diffusion again appears to be very limited.

### Comparison of IMC evolution in Co/Sn and Cu/Sn systems

As shown in Fig. 1, two different IMC growth behaviors and morphologies are found for the Co/Sn and Cu/Sn systems. The IMC evolution of CoSn_3_ is uniform and there is a limited impact from the Sn grain boundaries. For the Cu_6_Sn_5_ IMC phase, the Sn grain boundaries have a significant effect on the morphology. An impact of Sn grain size on the growth kinetics of the IMC layers was also found in experiments for the Cu/Sn system, in agreement with the findings from our simulations for Group 2. Zhao *et al*. investigated the effect of Sn grain size on the IMC morphology and evolution of Cu_6_Sn_5_ IMC phase during solid state reaction of Cu/Sn at 150 °C^21^. The results indicate that the IMC layer tended to be thicker for smaller Sn grain size. It was also clearly seen that the Cu_6_Sn_5_ preferentially grew along the Sn grain boundaries, in agreement with the observations for the simulation group 2, which is performed for a α ratio smaller than 1. Combined with the insights obtained from the simulation results of Group 1 and 2, it can be inferred that the non-uniform IMC morphology, as found for Cu/Sn solid state reaction, indicates that *L*_*gb*_ > *L*_*int*_, with *L*_*int*_ related to the interface mobility of the Cu_6_Sn_5_ IMC/Sn interface and *L*_*gb*_ related to the grain boundary mobility of the Sn grain boundaries. The observed impact from Sn grain size on the IMC growth rate of the Cu_6_Sn_5_ phase can then also be explained as a result of the relatively higher value for grain boundary mobility in the Sn phase compared to the mobility of the IMC/Sn interface. This hypothesis is verified by means of multi-grain inter-diffusion simulations for the Cu/Sn system.

To verify this hypothesis, a multi-grain simulation of Cu/Sn solid-state reaction is performed, using optimized input data for this system. The optimized parabolic Gibbs energy coefficients, equilibrium concentration and inter-diffusion coefficients used in the simulations for the Cu/Sn solid-state reaction are listed in Table 7. The parabolic Gibbs energy of each phase in the Cu/Sn system are formulated based on thermodynamic data extracted from the Thermo-Calc TCSLD3 database. The inter-diffusion coefficients *D* of the different phases in the Cu/Sn system are taken from the study of Yuan *et al*.^36^. Two IMC phases, Cu_6_Sn_5_ and Cu_3_Sn, are considered in the simulation domain. A polycrystalline grain structure for the Sn and IMC phases was generated to initialize the simulations. A simulation domain with 120 × 50 × 50 grid points is used. Other parameters like grid spacing, interfacial energy and molar volume were all taken as listed in Table 2. The value of *L*_*int*_ for Cu/Sn system used in the simulations is calculated from Eq. (12) to obtain diffusion-controlled growth. Based on our findings discussed in the previous sections, *L*_*gb*_ is taken to be 10* *L*_*int*_ and α equals 0.1. Since the value of the grain boundary diffusion mobility *M*_*gb*_ seems to have very limited impact on the IMC evolution and no experimental data are available for this parameter, *M*_*gb*_ = *M*_*bulk*_ is arbitrarily assumed in the simulations.

**Table 7:** The thermodynamic coefficients of the free energy densities, concentration of each phase and inter-diffusion coefficients D for Cu/Sn system considered in the simulations at 433 K.

Phases	*A* ^ρ^	*B* ^ρ^	*C* ^ρ^	*x*_0_^*ρ*^	inter-diffusion coefficients D^*ρ*^	*L*_*int*_	*L*_*gb*_
Cu	2.6 × 10^10^	−4.04 × 10^9^	−1.54 × 10^9^	0.022	1.1 × 10^−25^	1.2 × 10^−13^	1.2 × 10^−12^
Cu_3_Sn	2.6 × 10^11^	−4.04 × 10^9^	−2.54 × 10^9^	0.250	3.5 × 10^−17^		
Cu_6_Sn_5_	2.68 × 10^10^	5.02 × 10^8^	−2.63 × 10^9^	0.455	9.5 × 10^−17^		
Sn	1.68 × 10^13^	5.02 × 10^8^	−2.29 × 10^9^	0.999	1 × 10^−16^		

**Figure 10:**
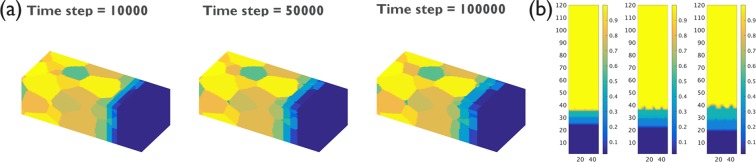
(**a**) 3-dimensional view of IMC evolution for Cu/Sn multi-grain simulation from time step 10000 to 100000; (**b**) cross-section representation of the evolution of the composition throughout the joint as obtained from phase-field simulation. A wavy interface develops for the Cu_6_Sn_5_ IMC phase. Scallops form at positions that correlate with the positions of Sn-grain boundaries.

The evolution of the two IMC phases Cu_3_Sn and Cu_6_Sn_5_ in the Cu/Sn simulations at time steps 10000 to 100000 are shown in Fig. 10(a). A cross-section of the composition profile is also shown in Fig. 10(b). It can be seen that the Cu_6_Sn_5_ phase preferentially grew along the Sn grain boundaries, which confirms the discussion in the last section. The conclusions from the last section thus also apply to the solid-state reactions for the Cu/Sn system with two IMC phases.

## Conclusions

In this paper, a phase field model for Sn-based solder and Co substrate is developed to predict IMC evolution at a specific temperature. A systematic investigation of the effect of the interfacial mobility, grain boundary mobility_,_ grain boundary diffusion coefficient and Sn grain structure on the IMC growth kinetics and morphology was performed. Several key conclusions can be drawn and demonstrated from the simulation results:

- Higher grain boundary diffusion *M*_*gb*_ leads to thicker IMC layer. Grain boundary diffusion *M*_*gb*_ has limited impact on the IMC growth kinetics and IMC morphology, while the interfacial mobility of the CoSn_3_/Sn interface and grain boundary mobility of the Sn grain boundaries have considerable impact. From the phase field simulations, it can be inferred that the ratio α = *L*_*int*_*/L*_*gb*_ is a key parameter determining the IMC morphology.
- The different morphologies found in the simulations resemble well the IMC morphologies observed in experiments for Co/Sn and Cu/Sn solid-state diffusion couples. A uniform IMC formation is found for Co/Sn systems. Based on our simulation study, we can conclude that this indicates that the mobility of the grain boundaries in the Sn phase is relatively low or of a similar magnitude as the interfacial mobility of the CoSn_3_/Sn interface (i.e. for α = *L*_*int*_*/L*_*gb*_ = 1 in the simulations). The wavy morphology or non-uniform IMC growth seen in Cu/Sn systems indicates that the mobility of the Sn grain boundaries is relatively high compared to the mobility of the Cu_6_Sn_5_/Sn interface (i.e. for α = *L*_*int*_*/L*_*gb*_ = 0.1 and 0.01 in the simulations).
- Sn grain size has an impact on the IMC growth kinetics and morphology under specific conditions, namely when the mobility for the Sn grain boundaries is relatively higher than the mobility of the IMC/Sn interface (α < 1 in the simulations). The simulation results are consistent with the experiments on Cu/Sn solid state reaction performed by Zhao *et al*.^21^.

These simulation results indicate that the Sn grain structure in a solder joint, and thus the interfacial reaction between UBM and solder, can be affected by modifying the electroplating process of Sn during the bump fabrication stage. The impact of the variation of Sn grain structure will change for different systems such as Cu/Sn and Co/Sn. The miniaturization of the solder balls might also affect the kinetic reaction rate of IMC formation and morphology since the number and location of Sn grain boundaries in the solder can be expected to vary with solder joint size^37,38^. Our study shows that the impact of the shrinkage of the solder joints needs to be further investigated experimentally as well as using simulation for different metallurgical systems.

## Supplementary information

Appendixes

## Data Availability

The data that support the findings of this study are available from the corresponding author upon reasonable request.
