# Supplementary material for: Study of the effect of Sn grain boundaries on IMC morphology in solid state inter-diffusion soldering
Source: Sci Rep. 2019 Oct 16;9:14862. doi: 10.1038/s41598-019-51179-9 (PMC6795889; doi:10.1038/s41598-019-51179-9)
Supplement: Supplementary file 1 — Appendixes [file 41598_2019_51179_MOESM1_ESM.docx]

**Study of the effect of Sn grain boundaries on IMC morphology in solid state inter-diffusion soldering**

***Lin Hou^a,b^, Nele Moelans^a^*********, Jaber Derakhshandeh^b^, Ingrid De Wolf^a,b^, Eric Beyne^b^***

***^a^****Dept. Materials Engineering, KU Leuven, Leuven, Belgium*

***^b^****IMEC, Kapeldreef 75, 3001 Leuven, Belgium*

*Correspondence and requests for materials should be addressed to N.M. (email: nele.moelans@kuleuven.be)*

**Appendix A: Construction of Gibbs energy functions with parabolic composition dependence based on thermodynamic calculations**

The schematic overview of the approach applied to estimate the kinetic growth rate and morphology of the formed IMC based on the phase-field method is shown in Fig. A1. The thermodynamic parameters, which represent the thermodynamic properties of the phases present in the diffusion couple by constructing the functions of parabolic Gibbs energy, can be calculated from the existing thermodynamic database. With input the experimentally determined interdiffusion coefficients from literature and assumed interfacial energy, the IMC growth rate from phase field simulations can be compared with experimentally determined IMC growth rates. The results can be further used to optimize the thermodynamic parameters and obtain insight into the effects of various input parameters on IMC evolution behavior.


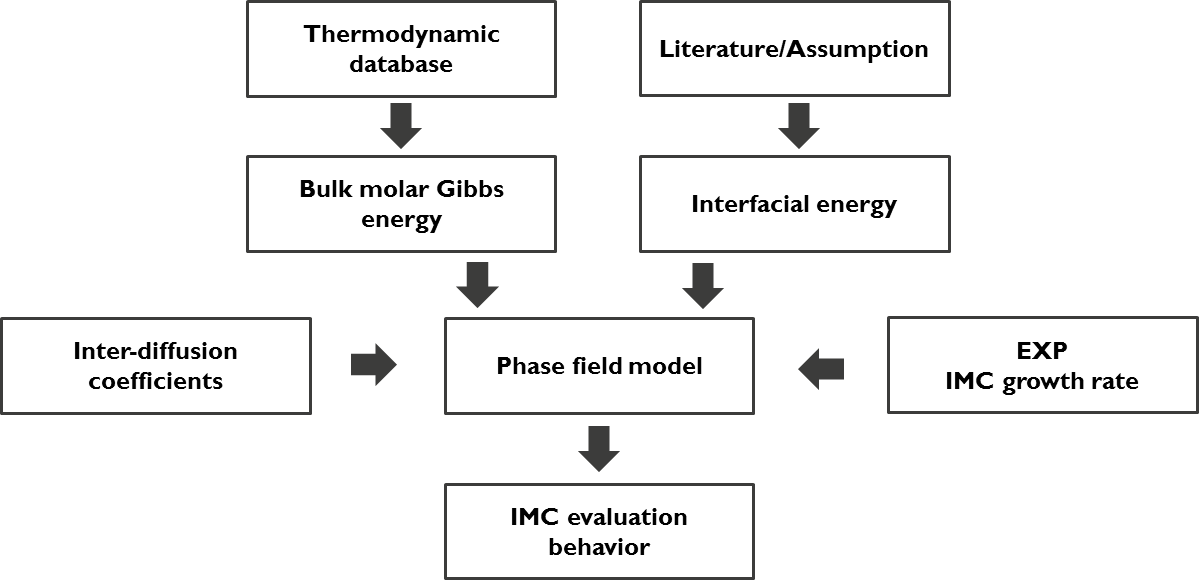


Fig. A1 Schematic overview of the applied method used to estimate the kinetic growth rate and morphology of IMC based on phase-field simulations in combination with experimental information.

As shown in Guan and Moelans work [1], the use of the parabolic Gibbs energy for the thermodynamic properties and phase diagrams has several advantages for the intended study. The complexity and computational cost to evaluate the bulk contribution in the free energy are largely reduced. The parabolic Gibbs energy of the phases ρ can be defined to be

$$\begin{aligned} f=\frac{A}{2}\left( x_{Sn}-x_{0}^{\rho} \right)^{2}+B\left( x_{Sn}-x_{0}^{\rho} \right)+C\#\left( A.1 \right) \end{aligned}$$

where $x_{Sn}$ is the molar fraction of Sn in phase ρ. The thermodynamic coefficient $A{,B}$ and $C$ are used to represent the thermodynamic properties of phase ρ.

According to the phase diagram of Co-Sn [2], two IMC phases are expected to be observed at the interface between Co UBM and Sn soldering during solid-state reaction, namely CoSn_2_ and CoSn_3_. However, only CoSn_3_ is detected at the interface of the Co/Sn diffusion couple in most experiments. The absence of the CoSn_2_ phase could imply that the inter-diffusion coefficient and solubility range of the CoSn_2_ phase is too small or that there is a high nucleation barrier for the formation of this phase during the solid-state reaction [3]. To simplify the simulation, only CoSn_3_ is considered in the present work. The molar volume V_m_ is assumed to be a constant value 10^5^ m^3^ mol^-1^ for all phases. The parameters $B$ and $C$ for the end phases hcp-Co and bct-Sn were then obtained as:

$$\begin{aligned} B=\frac{1}{V_{m}}\frac{\partial G_{m}}{\partial x}|_{{x_{0}}^{\rho}}=\frac{1}{V_{m}}\tilde{}|_{{x_{0}}^{\rho}}\#\left( A.2 \right) \end{aligned}$$

$$\begin{aligned} C=\frac{1}{V_{m}}\tilde{G_{m}}|_{{x_{0}}^{\rho}}\#\left( A.3 \right) \end{aligned}$$

with $\tilde{}|_{{x_{0}}^{\rho}}={\mu_{Sn}|}_{{x_{0}}^{\rho}}$-${\mu_{Co}|}_{{x_{0}}^{\rho}}$, with ${\mu_{Sn}|}_{{x_{0}}^{\rho}}$and ${\mu_{Co}|}_{{x_{0}}^{\rho}}$ are the chemical potentials of the elements Sn and Co in phase *r* at the composition given by $x_{Sn}=x_{0}^{\rho}$, respectively. $\tilde{G_{m}}|_{{x_{0}}^{\rho}}$ is the Gibbs energy evaluated at $x_{Sn}=x_{0}^{\rho}.$

The parameter $A$ is the second derivative of the Gibbs energy with respect to the mole fraction of Sn, which can be calculated by a finite difference:

$$\begin{aligned} A=\frac{1}{V_{m}}\frac{\partial^{2}G_{m}}{\partial^{2}x}|_{x_{0}}=\frac{1}{V_{m}}\frac{\partial\tilde{}}{\partial x}|_{x_{0}}=\frac{1}{V_{m}}\frac{\tilde{}\left( x_{0}+\delta x \right)-\tilde{}\left( x_{0}-\delta x \right)}{2\delta x}\#\left( A.4 \right) \end{aligned}$$

and $\delta x$ is taken to be 10^-6^.

The thermodynamic database used for the construction of Gibbs energy functions for Co/Sn system in the present work is based on the optimized Co/Sn phase diagram of Jiang et al [4]. For the CoSn_3_ phase, the Gibbs energy was considered to be independent with the composition in the thermodynamic database. The value for $C^{CoSn3}$can be extracted by calculating the Gibbs energy of the stoichiometric phase CoSn_3_ at the considered temperature for the thermodynamic database. The parameters $A^{CoSn3}$ and $B^{CoSn3}$cannot be obtained from the thermodynamic database and their values are optimized to obtain the experimentally obtained growth rate coefficient for the CoSn_3_ phase in the simulations, as described in section 4 in the paper.

For the end phases hcp-Co and bct-Sn, the phase composition *x^ρ^* was equal to the equilibrium molar fraction of Sn in phase ρ. For CoSn_3_ phase, the molar fraction of Sn in the IMC phase is taken equal to the stoichiometric composition of IMC, i.e. 0.75. In order to obtain a reasonable equilibrium concentration for the end phases, the thermodynamic coefficients, $A^{CoSn3}$ and $B^{CoSn3}$, of parabolic Gibbs energy densities are slightly modified from the initial values from the Co/Sn phase diagram database by applying the common tangent. The plot of parabolic Gibbs energies of all existing phases with the Sn molar fraction in the Co/Sn diffusion couple at 473 K are shown in Fig.A.2. The optimized thermodynamic values for phase field simulation are listed in Table 1.


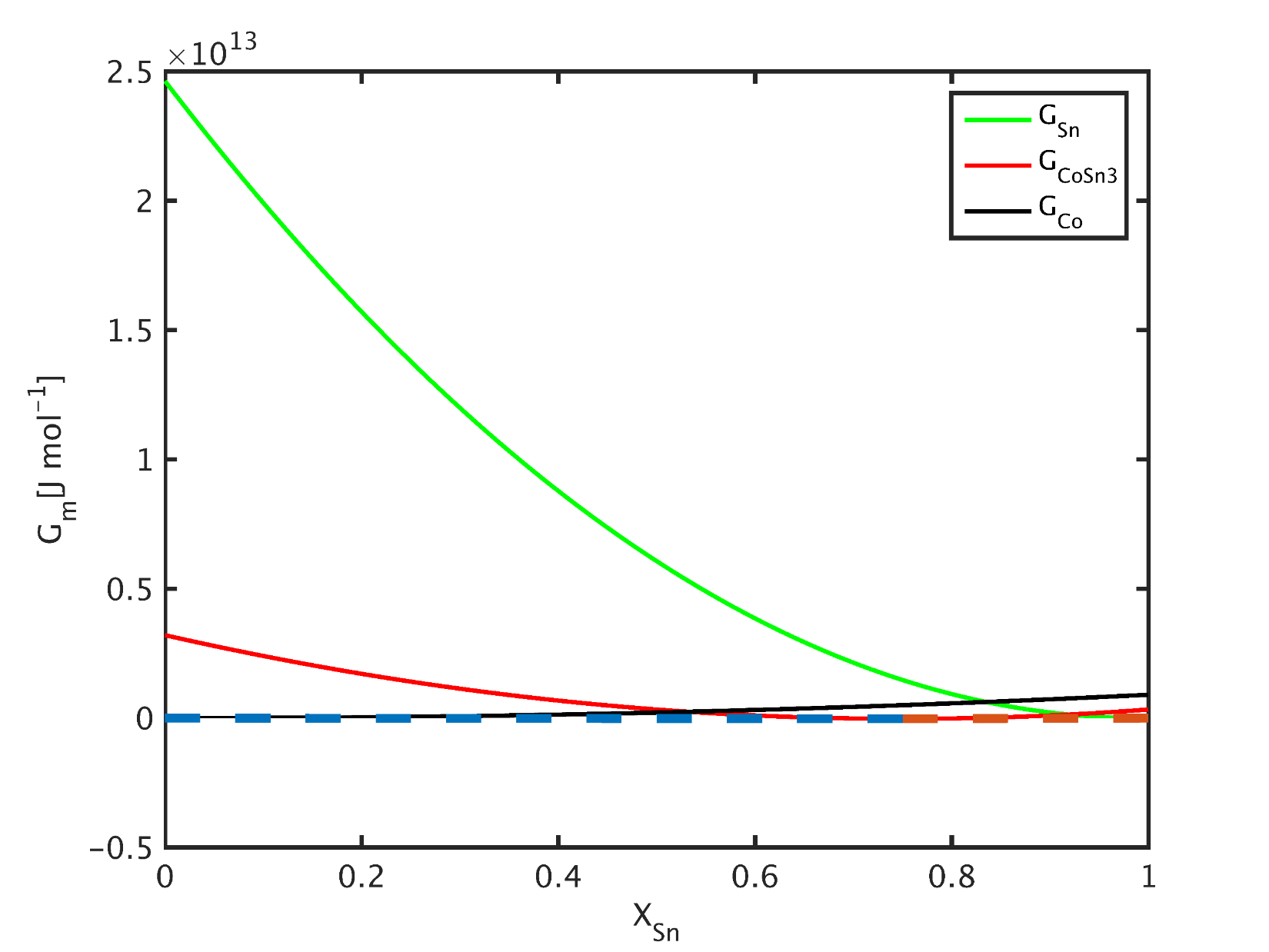


Fig. A.2 The plot of parabolic Gibbs energies of all existing phases with the Sn molar fraction in the Co/Sn diffusion couple at 473 K. Applying the common tangent construction, the equilibrium compositions for each phase can be obtained from the parabolic Gibbs energy functions, which are listed in Table 1.

**Appendix B: Inter-diffusion coefficients**

Wagner’s method [5] was used to extract the inter-diffusion coefficients from concentration profiles of Co-Sn solid state inter-diffusion couple with the equation:

$$\begin{aligned} Y\left( X \right)=\frac{N_{i}\left( X \right)-N_{i}^{+\infty}}{N_{i}^{+\infty}-N_{i}^{-\infty}}\#\left( B.1 \right) \end{aligned}$$

where $N_{i}\left( X \right)$ is the molar fraction of $i$ at the position $X$ and$N_{i}^{+\infty}\mathrm{and}N_{i}^{-\infty}$are the corresponding values in the left and right boundaries of the diffusion couple, respectively[8]. The inter-diffusion coefficient used in the present work is extracted from the molar fraction of phase *i* for Co/Sn diffusion coupl from EPMA (Electron probe micro-analyzer) measurement, which is taken from experiments of Takamatsu et al. [3]. High purity polycrystalline Sn (with purity of 99.99%) and Co substrate (with purity of 99.99%) is used in the experiment. The formed IMC morphology indicate very flat and uniform morphology, similar to the IMC morphology shown in Fig. 1(a) in the paper. The inter-diffusion coefficient *D^ρ^* of IMC phase can be extracted to be 4.73$\times{10}^{14}$ m^2^ s^-1^ under 473K from the results of Takamatsu et al. It should be mentioned here that the inter-diffusion coefficients for binary diffusion joints during solid state reaction can be affected by the fabrication process, since material purity, grain size and porosity *etc.* can vary [6][7][8]. To minimize the impact from material purity, the IMC inter-diffusion coefficient *D^ρ^*, from high purity diffusion couples of experiments of Takamatsu et al. is used for the present simulations. The simulated kinetic growth rates in the simulations presented in section 4 are compared with the experimental growth rates from Takamatsu et al, which are listed in Table 3. A more detailed discussion and calculation of the inter-diffusion coefficient from molar fraction profiles for binary diffusion couples can be found in Yuan et al. [9].

It’s assumed that inter-diffusion coefficients of the end phases Hcp-Co and Bct-(Sn) are composition independent. In the study of Guan et al [1], it was demonstrated that the interdiffusion coefficients of end phases, in this case Hcp-Co and Bct-Sn, have very limited effect on the IMC evolution behavior if the initial compositions of phases are equilibrium compositions. Since there is very limited study can be found for the inter-diffusion coefficient of Hcp-Co, a value$1\times{10}^{25}$m^2^ s^-1^, which is the inter-diffusion coefficient used for a Cu UBM in Cu/Sn diffusion couple, is used in Co/Sn simulations. For the Bct-(Sn) phase, a value of 1$.7\times{10}^{14}$ m^2^ s^-1^ was used for all Co/Sn simulations to allow a stable simulation with a larger time step. And the IMC evolution can be observed within a reasonable amount of time step.

**Appendix C: simulation result without grain boundary diffusion *M_gb_***


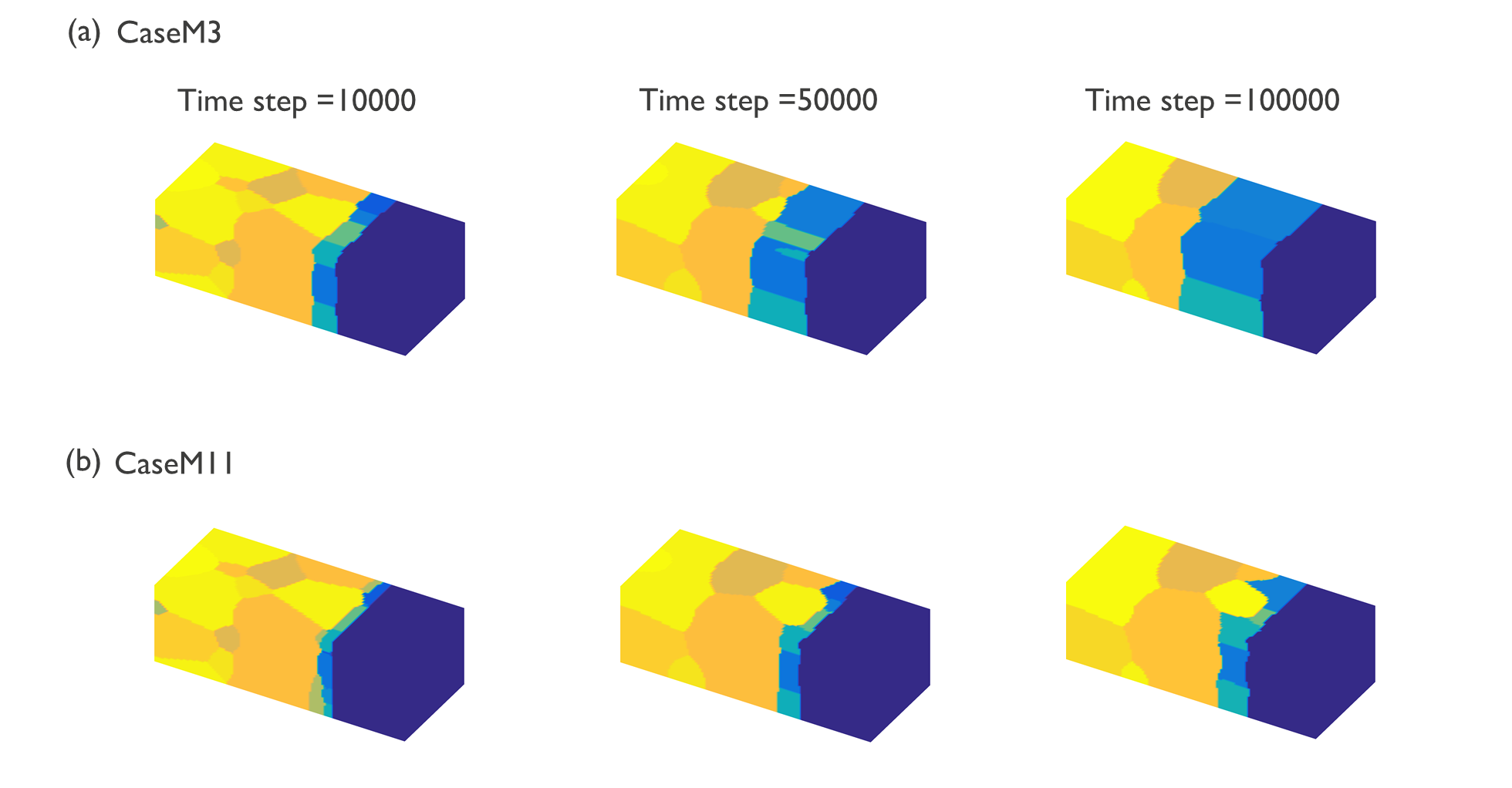


Fig. A.3(a)3-dimensional view of IMC evolution for Co/Sn multi-grain simulation from time step 10000 to 100000 for CaseM3;(b) 3-dimensional view of IMC evolution for Co/Sn multi-grain simulation from time step 10000 to 100000 for CaseM11;

**Reference**

1. Guan Y, Moelans N. Influence of the solubility range of intermetallic compounds on their growth behavior in hetero-junctions[J]. Journal of Alloys and Compounds, 2015, 635: 289-299
2. A. Lang, W. Jeitschko, Two new phases in the system cobalt-tin: the crystal structures of α- and β-CoSn3, Z. Metallkd. 87 (1996) 759-764.
3. Takamatsu Y, Kajihara M. Kinetics of Solid-State Reactive Diffusion between Co and Sn[J]. Materials Transactions, 2014, 55(7): 1058-1064.
4. Jiang, M., Sato, J., Ohnuma, I., Kainuma, R., & Ishida, K. (2004). A thermodynamic assessment of the Co-Sn system. Calphad, 28(2), 213-220.
5. Darken, L. S. Diffusion, mobility and their interrelation through free energy in binary metallic systems. Metall. Mater. Trans. A Phys. Metall. Mater. Sci. (2010). doi:10.1007/s11661-010-0177-7
6. R. Labie, W. Ruythooren, J. Van Humbeeck, “Solid state diffusion in Cu-Sn and Ni-Sn diffusion couples with flip-chip scale dimensions” Intermetallics 15 3 (2007) 396-403.
7. Y.H. Lee, H.T. Lee, “Shear strength and interfacial microstructure of Sn-Ag-xNi/Cu single shear lap solder joints” Materials Science and Engineering A 444 1-2 (2007) 75-83.
8. S. J. Wang, C.Y. Liu, “Study of interaction between Cu-Sn and Ni-Sn interfacial reactions by Ni-Sn3.5Ag-Cu sandwich structure” Journal of Electronic Materials 32 (2003)1303-1309.
9. Yuan Y, Guan Y, Li D, et al. Investigation of diffusion behavior in Cu–Sn solid state diffusion couples[J]. Journal of Alloys and Compounds, 2016, 661: 282-293
